# Application of a cationic amylose derivative loaded with single‐walled carbon nanotubes for gene delivery therapy and photothermal therapy of colorectal cancer

**DOI:** 10.1002/jbm.a.37351

**Published:** 2022-01-07

**Authors:** Zechang Chen, Junbo Zhuang, Jiadong Pang, Zehao Liu, Penghao Zhang, Haijun Deng, Liming Zhang, Baoxiong Zhuang

**Affiliations:** ^1^ Department of General Surgery & Guangdong Provincial Key Laboratory of Precision Medicine for Gastrointestinal Tumor Nanfang Hospital, Southern Medical University Guangzhou China; ^2^ The First School of Clinical Medicine Southern Medical University Guangzhou China; ^3^ Institute of Polymer Science, Department of Polymer and Materials Science, School of Chemistry and Chemical Engineering Sun Yat‐Sen University Guangzhou China

**Keywords:** amylose derivative, colorectal cancer, gene delivery, photothermal therapy, single‐walled carbon nanotubes

## Abstract

Single‐walled carbon nanotubes (SWNTs) are cylindrical graphitic helix molecules that exhibit superb mechanical and physical properties. Many polymers, such as polyethylene glycol and glycated chitosan, have been used to modify SWNTs to enhance the stability and biocompatibility of delivery systems; thus, a novel modification for SWNTs with amylose derivatives containing poly(L‐lysine) dendrons (ADP@SWNT) is developed. Infrared spectra analysis, ^1^H NMR analysis, circular dichroism spectra analysis and thermogravimetric analysis are used to characterize and confirm complex formation. The aqueous dispersion stability, cytotoxicity, gene transfection efficiency and photothermal effect of the complex are studied in vitro and in vivo. Results suggest that the ADP@SWNT complex is successfully synthesized with good water dispersion stability and pDNA transfection capacity. ADP@SWNT/TNFα inhibits tumor growth and metastasis both in vivo and in vitro, and the anti‐tumor effect is enhanced by NIR irradiation, suggesting its high potential for application in tumor therapy.

## 1 INTRODUCTION

Single‐walled carbon nanotubes (SWNTs) are cylindrical graphitic helix molecules that have been increasingly studied due to their superb mechanical and physical properties.^1^ They have been explored as delivery vehicles for cytotoxic drugs,^2^, ^3^, ^4^ proteins^5^ and nucleic acids.^6^, ^7^ In addition, SWNTs may be heated and exhibit photothermy in the near‐infrared (NIR) region,^1^, ^8^, ^9^ which allows their possible application in photothermal and photoacoustic therapy. Kang et al.^10^ reported that under irradiation with a 1064‐nm Q‐switched millisecond pulsed laser, SWNTs could generate a shockwave that resulted in mechanical damage to cancer cells. Additionally, Zhou et al.'s^11^ research team demonstrated that functionalized SWNTs were able to localize in the mitochondria of normal and cancerous cells. Consequently, under pulsed laser irradiation, the mitochondria‐localized SWNTs killed cancer cells mainly by triggering cell apoptosis through mitochondrial depolarization and the subsequent release of cytochrome C.^12^ However, cytotoxicity and limited dispersion stability of these SWNTs have largely impeded their application.^13^, ^14^ Furthermore, the length and surface chemistry properties of these SWNTs affect their cytotoxicity.^15^ In other words, the biocompatibility of SWNTs may be engineerable.^16^, ^17^, ^18^ Therefore, establishing biocompatible water‐soluble SWNTs for clinical application is highly necessary.

Tremendous efforts have been made to investigate nonviral gene delivery—cationic polymers, such as poly(L‐lysine), polyethyleneimine, and polymethacrylate.^19^, ^20^ Although these nonviral vectors provide benefits, including a low immune response, capacity to deliver large DNA molecules and low cost, they are still limited by their cytotoxicity, low stability, and targeting.^21^ Amylose is a natural polysaccharide that is made up of D‐glucosyl units connected by α‐(1,4) glucosidic bonds. Amylose molecules typically consist of 200–20,000 glucose units that form a helix. Due to their outstanding transparency, flexibility, stretching force and water insoluble nature, amylose molecules have been widely used in different fields.^22^, ^23^, ^24^

We have reported the chemical modification of amylose by click conjugation with the propargyl focal point poly(L‐lysine) dendron of the third generation (PLLD‐G3). The modified amylose derivative poly(L‐lysine) dendron (ADP) exhibited excellent ability to deliver plasmid DNA (pDNA) with less cytotoxicity and comparable transfection efficiency.^25^ Here, we carried out for the first time the synthesis of SWNTs functionalized with ADP (ADP@SWNT) and studied the complex regarding its water dispersion stability, cytotoxicity, gene transfection efficiency and in vitro and in vivo photothermal effects (Supplementary Figure 1).

## 2 MATERIALS AND METHODS

### 2.1 Materials

PLLD‐G3 was synthesized in our laboratory by divergent and convergent approaches, and ADP was synthesized in our laboratory by the click reaction between azidized amylose (Amy‐N3) and PLLD‐G3 as reported in our previous publication.^25^, ^26^ Raw SWNTs with lengths of 1 ~ 3 μm and diameters of 1 ~ 2 nm were obtained from Chengdu Organic Chemicals Co., Ltd., Chinese Academy of Sciences (Chengdu, China). Plasmids encoding tumor necrosis factor‐alpha (TNF‐α) and enhanced green fluorescent protein (EGFP)‐pIRES2‐EGFP‐TNF‐α were purchased from Santa Cruz Biotechnology Co. (Shanghai, China). Hoechst 33258 was purchased from Beyotime Biotechnology Co. (Shanghai, China). Horseradish peroxidase (HRP)‐conjugated anti‐rabbit and HRP‐conjugated anti‐mouse IgG were obtained from Golden Bridge International Inc. (USA). Dulbecco’s modified Eagle’s medium (DMEM), trypsin–ethylenediaminetetraacetic acid (trypsin–EDTA), and foetal bovine serum (FBS) were purchased from Gibco‐BRL (Canada). Ethidium bromide (EB) and 3‐[4,5‐dimethylthiazol‐2‐yl]‐2,5‐diphenyltetrazolium bromide (MTT) were obtained from Sigma, USA. The human colorectal cancer cell line HCT116 and human embryonic kidney 293 T cells were provided by Nanfang Hospital (Guangzhou, China). BALB/c nude mice were provided by the Centre of Experimental Animals of Southern Medical University. Other analytical grade chemical reagents were obtained from Guangzhou Chemical Reagent Factory (China) and were used directly.

### 2.2 Synthesis and structural characterization of ADP@SWNT

ADP@SWNT was synthesized by ultrasonic approaches. Briefly, 5 mg of SWNTs were placed in 10 mL of distilled water and sonicated using an ultrasonic probe (Sigma Ultrasonic Processor, GEX‐600). This process proceeded at 30 W for 20 min, during which each pulse lasted for 2 s followed by 2 s of rest. Sonication was repeated three times with a 10‐min suspension each time. Next, 10 mg of ADP was added to the above solution and sonicated for 30 min at 130 W, 50 Hz, and 50%. Similarly, the pulse width was 2 s (pulse period of 4 s), and this step was repeated three times with a 10‐min suspension each time. The resulting suspension was stirred for 12 h and centrifuged at 3000 r/s for 10 min, followed by filtration and lyophilization procedures. Finally, the inclusion complex, a PLLD‐G3‐based functionalized amylose derivative loaded with SWNTs, was obtained, with a yield of 47%. Infrared spectra were measured via the KBr squash method using an FTIR spectrophotometer (Nicolet 670, Thermo Nicolet Corporation, Wisconsin, USA). ^1^H NMR analyses (Mercury‐Plus 300 Varian, USA) for the PLLD‐G3, Amy‐N3, ADP and ADP@SWNT dispersions were used to confirm the formation of ADP@SWNT. Circular dichroism (CD) spectra measurements of the ADP, SWNTs, PLLD‐G3, ADP + SWNT and ADP@SWNT dispersions (1 mg/mL) were carried out using a J‐810 circular dichroism spectropolarimeter (Jasco, Easton, MD, USA).

### 2.3 Thermogravimetric analysis

A Netzsch TG‐209 thermobalance (NETZSCH, Germany) was employed for the thermogravimetric analysis (TGA) measurements. Samples were heated at 20°C/min from room temperature (30°C) to 800°C. Dry nitrogen served as both the sweep gas and protection gas at flow rates of 40 mL/min and 20 mL/min, respectively.

### 2.4 Water dispersion stability analysis

The SWNTs, ADP + SWNT and ADP@SWNT samples were dispersed in distilled water. The dispersion liquid was stirred for 30 min (the concentration of each sample was 1 mg/mL) and allowed to stand. A Shimadzu ultraviolet–visible (UV–vis) spectrophotometer was used to measure the absorbance at 500 nm of each dispersion liquid. SWNTs were dispersed in an ADP solution (1 mg/mL) by the ultrasonic dispersion method (Sigma Ultrasonic Processor, GEX‐600) (130 W, 50 Hz, 50%, 30 min). Subsequently, the suspension was centrifuged at 5000 rpm for different durations (0, 2, 5, 10, 20, and 30 min), and the centrifuged samples were measured at 500 nm by a Shimadzu UV–vis spectrophotometer.

Transmission electron microscopy (TEM) observation: A 20‐μL drop of SWNT solution and ADP@SWNT solution were placed on a 200‐mesh copper grid covered with a perforated carbon film and allowed to dry naturally for 60 s. The excess copper was drawn off gently with filter paper. The copper grid with samples on it was stained with 2% (w/v) phosphotungstic acid solution for 60 s, and filter paper was used to remove the residual solution. Then, the sample was imaged using TEM (JEM‐2010HR).

### 2.5 Photothermal effect

The photothermal effect of SWNTs was investigated under irradiation with a near‐infrared (NIR) laser. Briefly, 3 mg of SWNTs and ADP@SWNT were placed on two polyethylene (PE) plastic films. Then, the plastic films were exposed to continuous NIR laser irradiation at 808 nm for 10 s (1.4 W/cm^2^). Additionally, ADP@SWNT aqueous dispersions at four different concentrations were prepared and irradiated by a continuous laser at 808 nm (1.4 W/cm^2^). Then, the temperature at 0, 5, 10, 15, 20, and 25 min after irradiation of the dispersion was recorded.

### 2.6 Cell viability assays

Human embryonic kidney 293 T cells were cultured in DMEM supplemented with 10% FBS at 37°C, 5% CO_2_, and 95% relative humidity. The cells were seeded in 96‐well sterile flat‐bottom plates at an initial density of 1000–10,000 cells/well in 200 μL of growth medium (appropriate number of cells needed for each well was determined from growth curves) and incubated for 3–5 days to ensure adherence. Upon surface attachment, the cells were treated with ADP@SWNT solutions at various concentrations ranging from 0.1 to 6 (mg/ml). After incubation for 4 h, 20 μL of MTT solution at a concentration of 5 mg/mL was added to each well. The reaction product was solubilized with 150 μL of DMSO while stirring for 10 min. The absorbance (A490) was measured by a Benchmark Plus Microplate Spectrophotometer (BioRad, USA) at 490 nm. The cell viability (%) was calculated according to the following equation: cell viability (%) = [A490 (sample)/A490 (control)] × 100, where A490 (sample) was obtained in the presence of samples and A490 (control) was obtained in the absence of samples.

### 2.7 In vitro transfection

An ADP@SWNT/TNFα complex at an N/P ratio of 30 was prepared. Cells at the exponential phase from the human colorectal cell line HCT116 were plated in 6‐well plates at 2 × 10^5^ cells/well and incubated for 24 h. At 70% confluence, the cells were divided into three groups: the ADP group, ADP@SWNT/TNFα group and ADP@SWNT/TNFα+irradiation group. Then, the media were replaced with serum‐free DMEM containing the TNFα, ADP@SWNT/TNFα and ADP@SWNT/TNFα solutions (the filtration method was used for removing bacteria) and cultured at 37°C and 5% CO_2_ for 6 h. The ADP@SWNT/TNFα+irradiation group was irradiated by a continuous NIR laser at 808 nm (1.4 W/cm^2^) for 30 min, while the other served as a control without irradiation. Then, the media were changed to fresh media containing 10% serum. After a 48‐h incubation, an Olympus IX71 fluorescence microscope was used to observe the expression of TNFα in the cells.

### 2.8 In vitro cytotoxicity assay

To confirm that ADP@SWNT/TNFα complexes exerted genomic cell‐killing effects on HCT116 cells, an MTT assay was performed. Cells at the exponential phase from the human colorectal cancer cell line HCT116 were seeded in 96‐well plates at a density of 1 × 10^4^ cells/well in 200 μL of DMEM containing 10% FBS and cultured at 37°C in a 95% humidified atmosphere containing 5% CO_2_ in DMEM. After 12 h, the medium was replaced with serum‐free DMEM. The cells were divided into three groups: the ADP group, ADP@SWNT/TNFα group and ADP@SWNT/TNFα+irradiation group. Then, 200 μL of the ADP solution and ADP@SWNT/TNFα and ADP@SWNT/TNFα dispersions (1 mg/mL) were added to their respective groups. Particularly, after a 12‐h incubation, the ADP@SWNT/TNFα+irradiation group was exposed to NIR irradiation at 808 nm (1.4 W/cm^2^) for 30 s every 8 h and was repeated 3 times. After a total of 48 h of incubation for all groups, an Olympus IX71 fluorescence microscope was used to observe the expression of TNFα in the cells. Then, 20 μL of MTT (5 mg/mL) was added in each well. After an additional 4‐h incubation, the MTT‐containing medium was removed, and 150 μL of DMSO was added to each well, followed by 10 min of continuous shaking. The absorbance was measured using the same method as stated above.

### 2.9 Cellular apoptosis assay

After transfection, HCT116 cells were quickly trypsinized, detached from plastic plates, and washed twice with PBS. Then, the cells were suspended in N‐2‐hydroxyethylpiperazine‐N‐ethane‐sulfonic acid (HEPES) buffer and stained with Annexin V and propidium iodide for 15 min. The stained cells were assessed using a FACS Aria flow cytometer (Germany).

### 2.10 In vivo anti‐tumor therapy

HCT116 cells (2 × 10^6^) were injected subcutaneously into the right flank region of BALB/c mice to establish a model of colorectal cancer‐bearing mice. After 3 days, in which the tumors reached a diameter of approximately 0.1 cm, the mice were randomly divided into three groups of five mice: the ADP group, ADP@SWNT/TNFα group, and ADP@SWNT/TNFα+irradiation group. Then, the mice were injected with 200 μL of ADP, ADP@SWNT/TNFα or ADP@SWNT/TNFα (1 mg/mL) into the tumor in situ at day 5, 8, and 13. Additionally, mice in the ADP@SWNT/TNFα+irradiation group were exposed to continuous NIR laser irradiation (808 nm) for 10 s (1.4 W/cm^2^) on day 17. A thermal imaging system was used to record the real‐time temperature; additionally, the tumor size was measured and recorded every other day. The mice were sacrificed on day 19, and their tumors were extracted for histopathology observation. Hematoxylin and eosin (H&E) staining was conducted to analyze the ADP@SWNT/TNFα toxicity towards major organs, including the heart, liver, brain, lung, and kidney, after 7 days. The animal experiment was conducted under the guidance of the Centre of Experimental Animals of Sun Yat‐sen University. Histopathological observation and immunohistochemistry were conducted by the Department of Pathology, Sun Yat‐Sen Memorial Hospital. TEM was carried out in the TEM room at the Medical College of Sun Yat‐sen University.

## 3 RESULTS

### 3.1 Synthesis and characterization of ADP@SWNT

**FIGURE 1:**
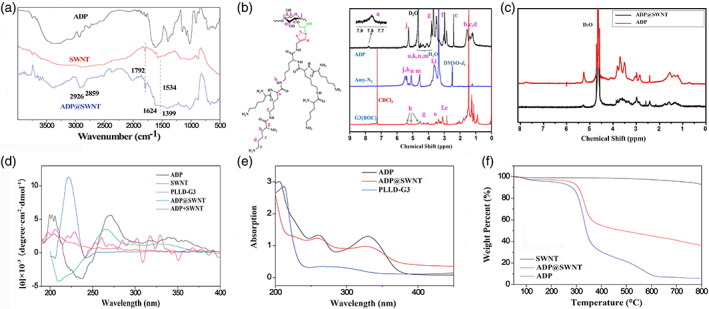
Synthesis and characterization of ADP@SWNT. (A) FTIR spectra of ADP, SWNTs, and ADP@SWNT. (B) Chemical structure of ADP (left), and the 1H‐NMR spectra of PLLD‐G3, Amy‐N3, and ADP (right). (C) 1H NMR spectra (in D2O) of ADP and ADP@SWNT. (D) CD spectra of ADP, SWNTs, PLLD‐G3, ADP+SWNT, and ADP@SWNT. (E) UV‐vis absorption spectra of ADP, ADP@SWNT and PLLD‐G3. (F) Weight‐temperature curves of the SWNTs, ADP@SWNT and ADP by thermogravimetric analysis

The procedure for the synthesis of ADP and the scheme for ADP@SWNT and the ADP@SWNT/TNFα complex are shown in Scheme 1. SWNTs were embedded in the ADP by ultrasonic approaches. The FTIR analysis showed that the absorption peak bands of ADP@SWNT at 2926 cm^−1^, 2859 cm^−1^ (νC—H), 1624 cm^−1^ (νC=O) and 1399 cm^−1^ (νCO—NH) were similar to that of ADP. The carboxyl stretching vibration at 1790 cm^−1^ and the C—C bending vibration at 1570 cm^−1^ were similar to those of the SWNTs. Therefore, the FTIR analysis indicated that SWNTs were successfully introduced to ADP (Figure 1A).

The chemical structure of ADP is shown in Figure 1B (left panel), and the ^1^H NMR spectra of PLLD‐G3, Amy‐N3, and ADP are shown in Figure 1B (right panel). Regarding the ^1^H NMR spectra of ADP‐G3, the proton resonance signal of PLLD‐G3 was at *δ* = 1.10–1.60 ppm and 3.0–3.40 ppm, while the signal for the amylose glycosidic unit appeared at *δ* = 2.40–4.10 ppm, indicating that PLLD‐G3 was grafted to the main chain of Amy‐N3; thus, ADP was successfully synthesized. Elemental analysis suggested that the nitrogen content of ADP was 8.54%; thus, the substitution degree (defined as the quantity of grafted PLLD‐G3 per 100 glycosidic units) was 8.5. Figure 1C shows the ^1^H NMR spectra (in D_2_O) of ADP and ADP@SWNT. The characteristic peak appearing at 7.8 ppm was attributed to the proton of the pentazole ring that developed after the click reaction. Despite the characteristic peak at 8.0 ppm that could not be displayed, the proton peak of ADP@SWNT was almost the same as that of ADP.

The aqueous ADP@SWNT dispersion was characterized by CD spectroscopy. As shown in Figure 1D, the CD spectra of ADP were similar to those of ADP@SWNT‐both had one negative effect and two positive effects. However, the spectra of the SWNT and ADP + SWNT dispersions were cluttered. Therefore, simply mixing ADP with SWNTs led to their entanglement, and the irregular SWNTs in the dispersion subsequently affected the spatial configuration of ADP. In addition, the structure of the amylose segment in ADP@SWNT achieved by the sonication method had no significant change.

As shown in Figure 1E, ADP@SWNT had three characteristic peaks, which were identical to those of ADP. In addition, we detected a significant blueshift at 220 nm in ADP@SWNT that was probably caused by the interaction between the hydrophobic cavity of amylose and the conjugated π‐bond at the surface of the nanotubes. In addition, ADP@SWNT exhibited a decrease in the peak intensity at 330 nm. This result may be due to the weakened conjugated structure caused by the interaction between the ADP and SWNTs. Collectively, both the CD spectra and the UV–vis absorption spectra further confirmed the successful synthesis of SWNTs functionalized with ADP.

### 3.2 Thermogravimetric analysis

To evaluate the encapsulation of SWNTs by ADP, we studied the weight loss by TGA from 30 to 800°C, and the weight‐temperature curve for the system is shown in Figure 1F. The weight loss of the SWNTs was only 2.72%, which was mainly because of their volatilization. Thus, SWNTs did not experience thermal degradation from 30 to 800°C. However, the TGA degradation curves for ADP and ADP@SWNT were the same since both of them exhibited two obvious mass losses. The total mass loss was estimated to be 94.11% and 64.00% for ADP and ADP@SWNT, respectively. Thus, the ADP and SWNTs accounted for 67.05% and 32.95% of the system, respectively. Moreover, the described data indicated that the addition of SWNTs to ADP@SWNT led to less weight loss due to thermally degraded components. Thus, the results confirmed that we successfully introduced SWNTs to ADP.

### 3.3 Water dispersion stability of ADP@SWNT

**FIGURE 2:**
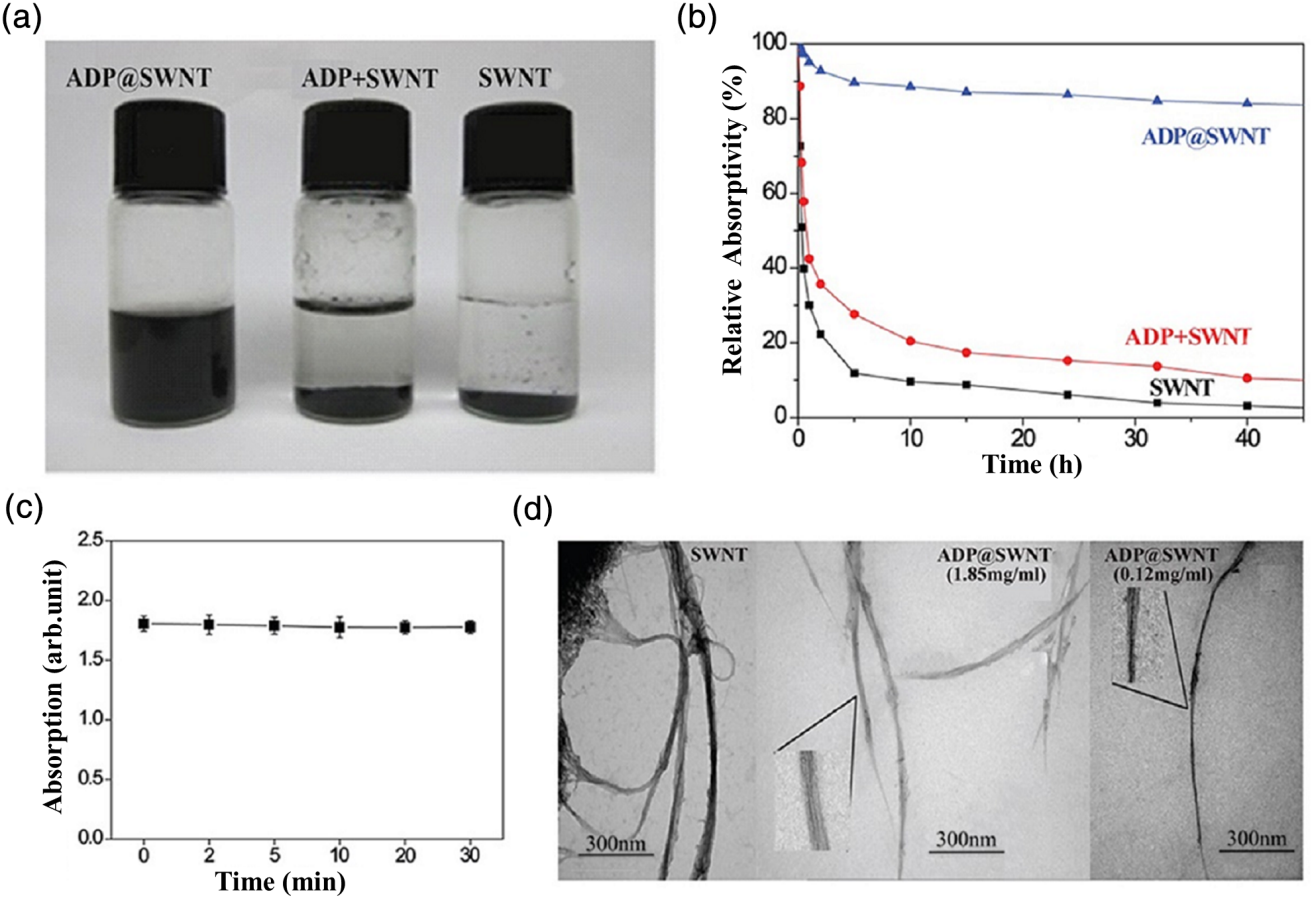
Water dispersion stability of ADP@SWNT. (A) Solubility of ADP, ADP+SWNT and ADP@SWNT in distilled water. (B) Stabilities of SWNTs (1 mg/ml) suspended in water, an SWNT dispersion in an ADP aqueous solution (1 mg/ml), and an ADP@SWNT aqueous solution (1 mg/ml) under centrifugation (5000 rpm). (C) Stabilities of ADP@SWNT under centrifugation (5000 rpm) for different times. Data were presented as the mean ± SD (*n* = 3)

As shown in Figure 2A, SWNTs have poor water solubility, agglomerating and depositing quickly in water. In contrast, ADP@SWNT exhibited better water solubility and remained stable for longer than 48 h in water. In addition, PLLD‐G3 showed a certain clath ratio for TNF‐a. As shown in Figure 2B, SWNTs aggregated after 5 h in aqueous solution, and the absorption of the dispersion decreased quickly at 300 nm. Similarly, the ADP + SWNT dispersion was found to precipitate quickly, while the ADP@SWNT retained their preferable water dispersibility, and the absorption at 300 nm decreased relatively slowly. Therefore, SWNTs were mainly encapsulated in the cavity of the amylose of ADP, which enhanced the water dispersibility of the nanotubes.

We further evaluated the aqueous dispersion stability of ADP@SWNT after centrifugation (5000 rpm, 30 min) (Figure 2C). The absorption of ADP@SWNT at 300 nm showed no significant change. Thus, the prepared dispersion solution could remain stable even with exogenous force.

TEM showed that the pristine SWNTs contained impurities such as amorphous carbons, nanoparticles, and metal catalysts. Thus, the SWNTs were twisted and frizzled, forming a cluster. However, the overlap of the SWNTs changed after dispersion in water, and singular SWNTs could be observed in the TEM image despite the presence of impurity particles. Additionally, the ADP@SWNT dispersion at a low concentration showed a layer of material attached to the sidewalls of the SWNTs (Figure 2D).

### 3.4 Photothermal effect of ADP@SWNT

**FIGURE 3:**
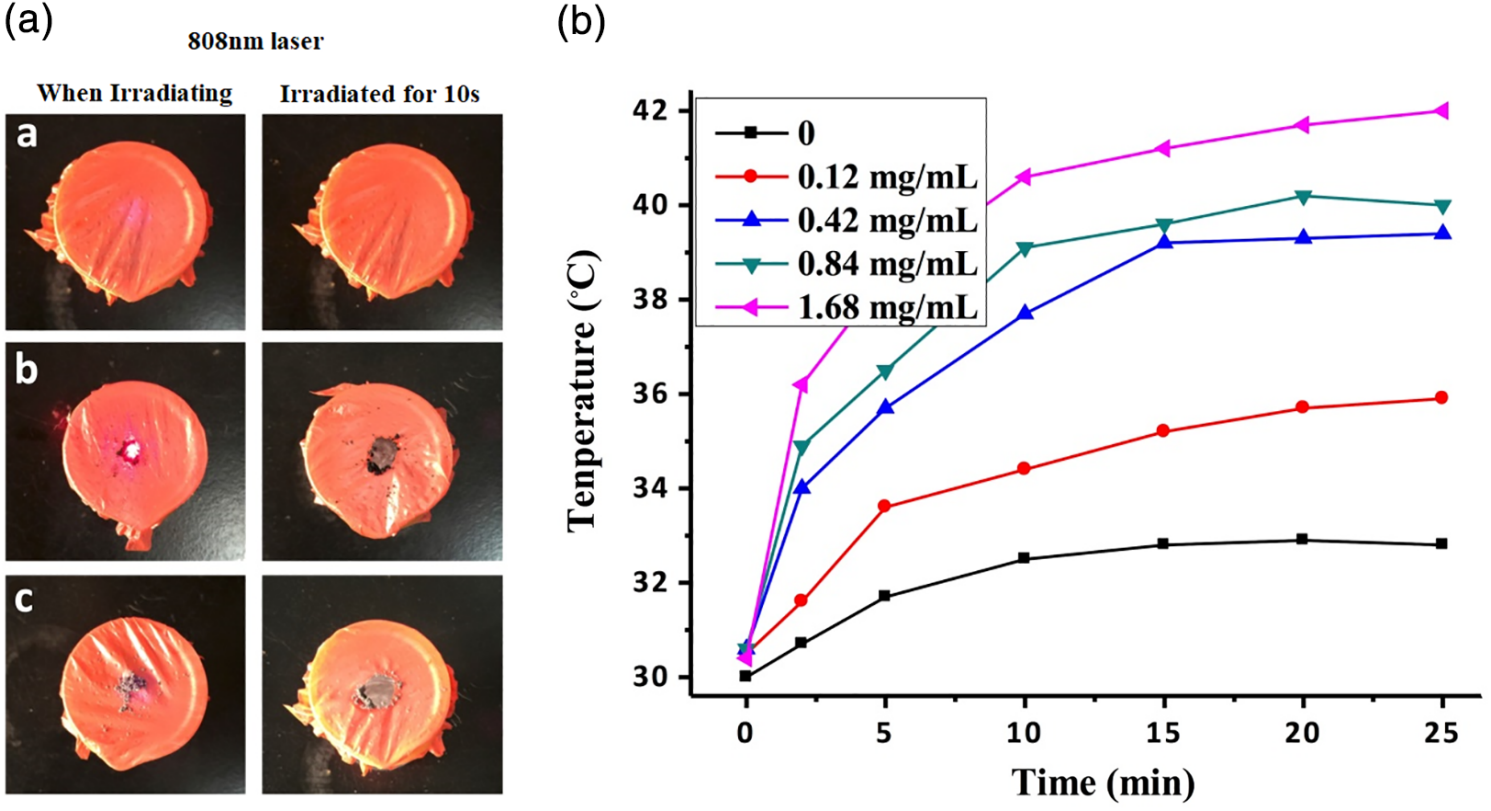
Photothermal effect of SWNTs and ADP@SWNT. (A) Photographs of the blank, SWNT powder‐loaded and ADP@SWNT powder‐loaded plastic films. (B) Temperature evolution for the aqueous ADP@SWNT dispersions at different concentrations under continuous irradiation by an 808 nm laser at 1.4 W/cm^2^

An in vitro photothermal effect analysis showed that there was no significant change in the empty plastic film before and after irradiation (Figure 3A). However, the plastic film burned after 10 s of laser irradiation when loaded with either SWNTs or ADP@SWNT (Figure 3A); that is, ADP@SWNT reserved a certain photothermal transformation ability similar to that of nanotubes. We further observed the temperature variation for aqueous ADP@SWNT dispersions at four different concentrations under continuous laser irradiation at 808 nm (1.4 W/cm^2^). As shown in Figure 3B, the photothermal transformation efficiency was associated with the irradiation duration and tended towards stabilization after approximately 10 min. In addition, the temperature of the aqueous dispersion at concentrations of 0.84 mg/mL and 1.68 mg/mL could reach over 40°C, which was sufficient to kill tumors at the cellular level. Overall, our results demonstrated that ADP@SWNT exhibited a strong photothermal transformation ability under NIR irradiation, which was promising for NIR‐triggered photothermal therapy.

### 3.5 Cell viability assays

**FIGURE 4:**
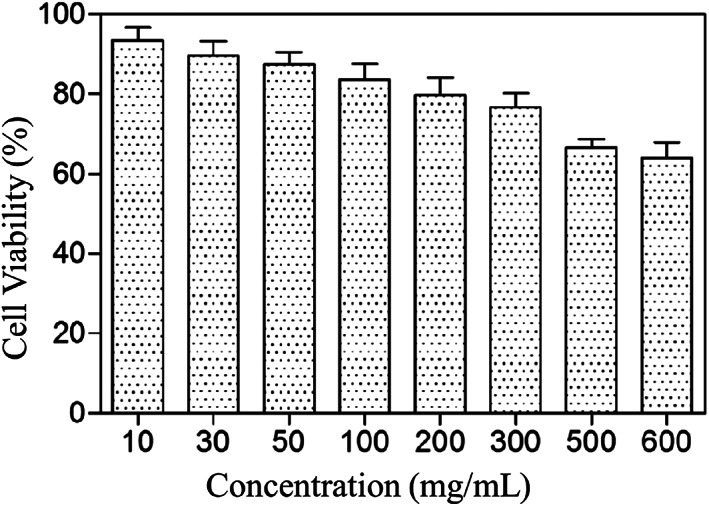
In vitro cytotoxicity of the ADP@SWNT dispersions at various concentrations. MTT assays for the in vitro cytotoxicity of different concentrations of ADP@SWNT dispersions (1 mg/ml) in 293T cells

The in vitro cytotoxicity of the ADP@SWNT dispersions at various concentrations ranging from 0.1 to 6 (mg/ml) was evaluated in 293 T cells by MTT assays. As shown in Figure 4, ADP@SWNT exhibited high cell viability, even at high concentrations. At a concentration of 1 mg/mL, for example, the cell viability was found to be approximately 86%. Clearly, the modified amylose displayed much lower cytotoxicity. These results were attributed to the good biocompatibility of ADP, which is preferable when this type of modified polysaccharide derivative is used for gene delivery.

### 3.6 pDNA‐binding ability

ADP@SWNT has an abundance of cationic charges, allowing its combination with negatively charged pDNA for the synthesis of a ADP@SWNT/TNFα complex. Gel electrophoresis was performed to assess the pDNA condensation ability of ADP@SWNT (Supplementary Figure 1). The migration of pDNA was notably slowed when the ADP/pDNA weight ratio exceeded 10. Thus, it could be concluded that ADP had good pDNA‐binding ability.

### 3.7 In vitro transfection of pDNA

**FIGURE 5:**
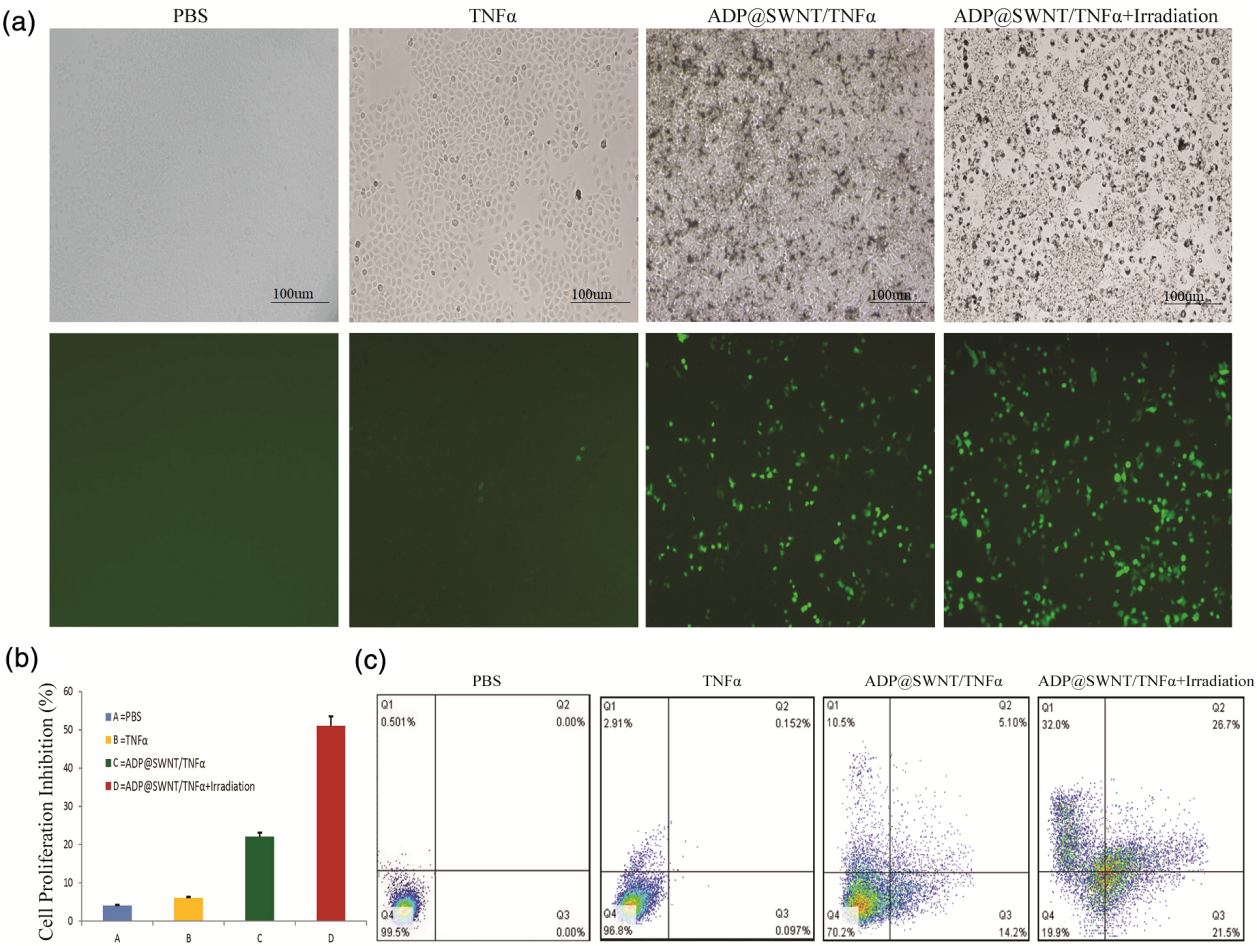
Bio‐assessment of ADP@SWNT/TNFα. (A) Fluorescence images of the HCT116 cells transfected with the plasmid: ADP@SWNT/TNFα (without irradiation) and ADP@SWNT/TNFα (with irradiation). (B) MTT assays (C) Cell apoptosis assay of PBS, ADP, ADP@SWNT/TNFα (without irradiation), and ADP@SWNT/TNFα (with irradiation)

As shown in Figure 5A, pDNA was effectively transfected into HCT116 cells and successfully expressed. Compared to the TNFα group, the pDNA transfection efficiency mediated by ADP@SWNT increased with higher GFP expression. We further studied whether extra irradiation would affect the transfection efficiency. The results showed that NIR irradiation promoted TNFα release from the ADP@SWNT/TNFα complex; thus, ADP@SWNT/TNFα exhibited higher transfection efficiency.

### 3.8 Proliferation inhibition on tumor cells

As shown in Figure 5B, no significant cell cytotoxicity of ADP was found, while the ADP@SWNT/TNFα complex exhibited an obvious inhibitory effect. Moreover, the ADP@SWNT/TNFα complex displayed a stronger inhibitory effect on HCT116 cells under NIR irradiation. Overall, our results indicated the valid inhibitory effect of NIR‐triggered ADP@SWNT/TNFα on tumor cells.

### 3.9 Cell apoptosis assay

The results of the cell apoptosis analysis are shown in Figure 5C. There was no significant cell apoptosis, and the apoptosis rate (including the early and late stages) was only 0.249% in the ADP group, which was similar to that of the MTT assay. However, HCT116 cells transfected with ADP@SWNT/TNFα experienced obvious apoptosis, with the apoptosis rate increasing to 19.3%. Similar to the MTT assay, ADP@SWNT/TNFα+irradiation had the best inhibitory effect, the apoptosis rate of which was 48.2% (Figure 5C). Collectively, our results indicated that functionalized SWNTs also killed tumor cells by triggering cell apoptosis. In addition, the apoptosis rate significantly increased after being triggered by NIR irradiation, which further confirmed the possibility and efficiency of genomic and photothermal therapy for cancer.

### 3.10 In vivo anti‐tumor

Before laser irradiation at 808 nm, the skin temperature of nude mice in the ADP group and ADP@SWNT group was approximately 35°C. During irradiation, the temperature in the ADP group increased extremely slowly with an approximately 10°C increase in 1–2 min. In contrast, the temperature in the ADP@SWNT group showed a quick increase of over 20°C in 1 min. In addition, the tumor tissue in the ADP@SWNT group became white and oedematous, suggesting that laser irradiation inhibited tumor growth because of the SWNTs.

**FIGURE 6:**
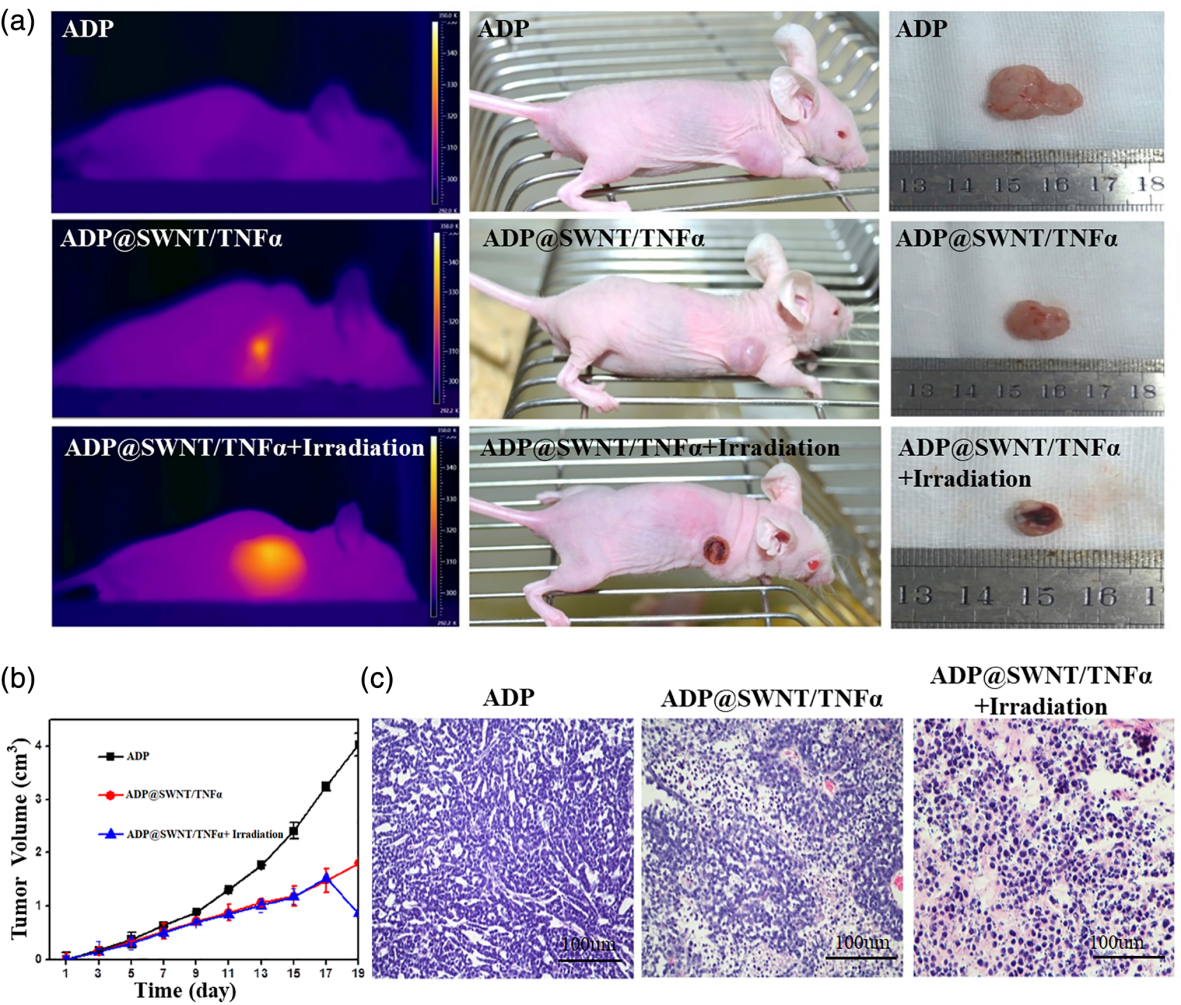
Tumor‐inhibitory effects of ADP@SWNT/TNFα and ADP@SWNT/TNFα+laser irradiation on human colorectal cancer in nude mice. (A) Photographs, (B) Tumor volumes (measured every 2 days) (C) Pathological examination of the gastrointestinal cancer tissues of nude mice injected with ADP, ADP@SWNT/TNFα, and ADP@SWNT/TNFα+irradiation

As shown in Figure 6A, no evidence of tumor necrosis was observed in the ADP group. Similarly, no remarkable damage was detected in the ADP@SWNT/TNFα group except that their shape became irregular. Regarding the ADP@SWNT/TNFα+laser irradiation group, a high scathe level was observed, and the tumor size decreased with heat ambustion on the skin. The results further demonstrated that the NIR‐triggered photothermal effect of ADP@SWNT/TNFα could effectively damage tumor cells, showing promise for its application in tumor therapy.

As shown in Figure 6B, the tumor size was similar among the three groups at the beginning. Shortly after 5 days, the tumor in the ADP group began to develop remarkably and was distinguishable from the tumors of the other two groups. At day 19, the tumor in the ADP group grew to 4 cm^3^, while the tumor in the ADP@SWNT/TNFα group was only 1.8 cm^3^. The anti‐tumor effect represented in the ADP@SWNT/TNFα group was due to the delivery of TNFα via the complex into the tumor. On the one hand, pDNA encodes TNFα, which inhibits tumor growth; on the other hand, the membrane penetrability of the SWNTs increases the damage to tumor cells. Regarding the ADP@SWNT/TNFα+irradiation group, since laser irradiation at 808 nm was applied at day 19, the tumor size was similar to that of the ADP@SWNT/TNFα alone group during the first 18 days. Notably, the tumor size for the ADP@SWNT/TNFα+irradiation group sharply decreased by more than 50% after treatment with irradiation, which indicated that NIR irradiation could effectively and quickly trigger the light‐heat conversion characteristics of ADP@SWNT/TNFα, thereby damaging the tumor.

**FIGURE 7:**
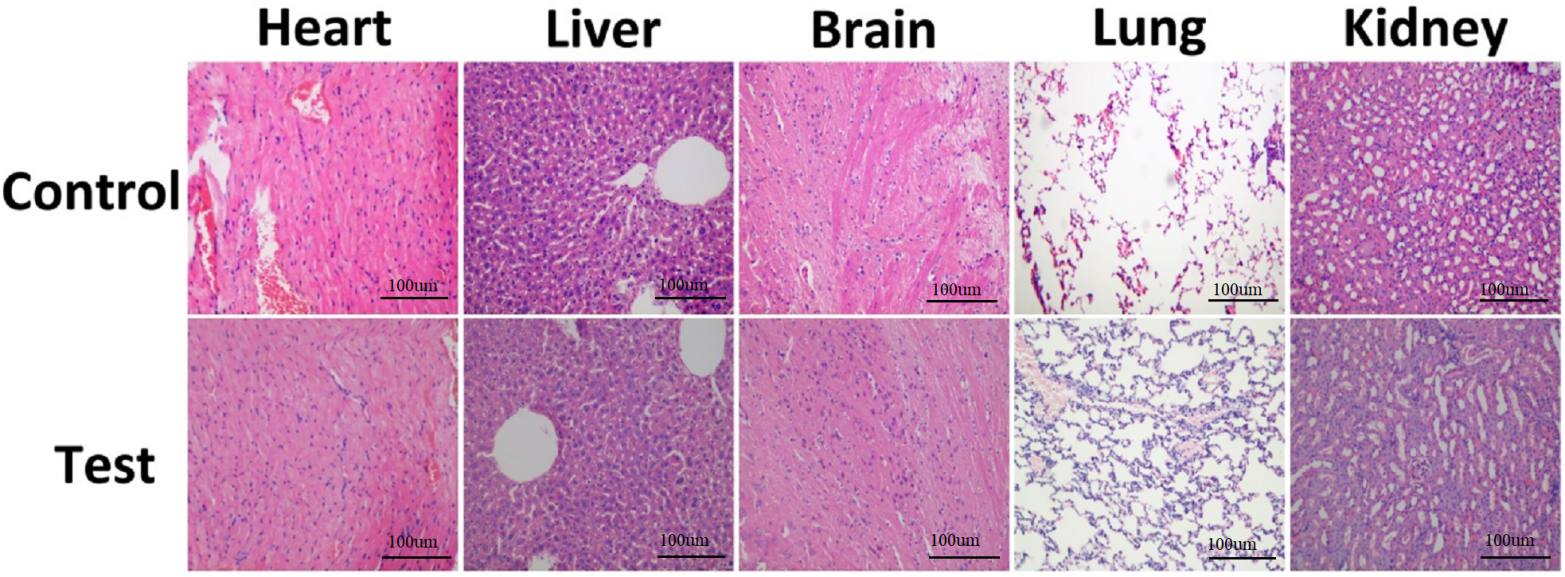
Pathological examination comparison of the major organs between the nude mice control group and nude mice injected with the ADP@SWNT/TNFα complex

As shown in Figure 6C, a disordered structure was observed in the colorectal cancer tissue of the ADP group, but the cancer cells remained complete and were generally in good status. Regarding the ADP@SWNT/TNFα group, the tumor structure was partially damaged, and cell necrosis could be found. In addition, regarding ADP@SWNT/TNFα+laser irradiation, the killing effect was greater, showing severe structural damage to the tumor and cell necrosis. In addition, compared with the control group, no significant toxicity was found among the major organs, including the heart, liver, brain, lung, and kidney (Figure 7). These results suggested that the ADP@SWNT/TNFα complex could effectively inhibit tumor growth and metastasis under NIR irradiation while showing no obvious toxicity to major organs.

## 4 DISCUSSION

A tremendous amount of research has been reported on the anti‐tumor effects of SWNTs in photoacoustic therapy, chemotherapy, thermoacoustic therapy and so on.^10^, ^12^, ^27^ However, the cytotoxicity and dispersion stability of SWNTs are still problems to solve before their application in biomedicine.^13^, ^14^ Polymeric materials have been widely used as gene delivery agents,^28^ and we have previously reported a modified amylose derivative that exhibits excellent ability to deliver pDNA with low cytotoxicity and comparable transfection efficiency.^25^ Here, we successfully synthesized an ADP@SWNT complex and characterized it by FTIR analysis, ^1^H NMR analysis, CD spectra analysis and TGA. The study demonstrated that, as predicted, the SWNTs were coated with ADP, forming a complex with good water dispersion stability and pDNA transfection capacity. In addition, we found that the ADP@SWNT/TNFα complex could inhibit tumor growth and metastasis both in vivo and in vitro and that the anti‐tumor effect was enhanced by NIR irradiation. The reason why ADP@SWNT/TNFα exhibited an obvious inhibitory effect was due to the SWNT‐induced penetration effect, which inhibited the proliferation of cancer cells. Additionally, with NIR irradiation, more pDNA was released, making it easier to destroy the DNA of cancer cells, and the photothermal effect of ADP@SWNT/TNFα also inhibited cell proliferation.

Since SWNTs have poor water solubility, in which they agglomerate and deposit quickly in water, achieving dispersion stability is one of the keys to the photothermal applications of SWNTs. To overcome the dispersion problem, methods for modifying carbon nanotubes mainly include covalent and non‐covalent methods. Covalent modification mainly use chemical approaches that involve the esterification and amidation of oxidized CNTs along with cycloaddition reactions to generate functional groups on the side walls. Regarding the non‐covalent approach, amphiphilic polymers, such as PEG,^29^, ^30^ are used to wrap or encapsulate the surface of carbon nanotubes.^31^, ^32^ In addition to disrupting the network structure, chemical approaches can lead to losses in the mechanical, electrical, and biosensing properties of SWNTs.^33^ In the present study, modified amylose encapsulated SWNTs in a non‐covalent manner without perturbing the network structure of the carbon nanotubes or changing their physical properties. In addition, PLLD‐G3 is an amphiphilic molecule that has been recognized as one of the most useful materials for functionalizing CNT surfaces to improve the dispersion of CNTs in aqueous media.^31^

ADP@SWNT is preferable in regard to its biotoxicity since amylose is biodegradable. In the present study, ADP@SWNT demonstrated a biocompatible profile suitable for gene delivery. In addition, our previous study suggested that modified amylose‐ADP displayed much lower cytotoxicity but considerable better gene delivery capability than polyethylenimine (bPEI)^,^ ^7^ a commonly used gene vector.^25^

Our study has some limitations. Although PLLD‐G3‐modified amylose enhances the DNA‐binding capacity of polylysine and increases the stability of nanocarriers, the efficiency of in vivo pDNA delivery is still a bottleneck. Factors associated with pDNA uptake include enzymatic degradation, rapid elimination by renal excretion or the mononuclear phagocyte system, poor cellular uptake and endosomal escape.^34^ Additionally, the specific pDNA uptake rate and TNFα expression rate of tumor cells are not reported in the present study. Furthermore, our in vivo experiment is limited by qualitative analysis, which is mainly based on morphological observations of the tumor‐bearing mice. Moreover, the in vivo metabolism, bio‐distribution and excretion of the complex are not discussed.

## 5 CONCLUSIONS

In this work, we demonstrated that SWNTs could be clathrated in ADP using the ultrasonication stirring method, effectively increasing the aqueous dispersity of SWNTs while retaining their photothermal conversion capacity. The ADP@SWNT/TNFα complex was proven to have good pDNA transfection ability, and NIR irradiation enhanced this transfection ability. In addition, the in vitro anti‐tumor analysis revealed that the ADP@SWNT/TNFα complex efficiently inhibited tumor growth under laser irradiation, suggesting synergy between the photothermal therapy and gene therapy. Similarly, the in vivo analysis showed the inhibitory effect of the ADP@SWNT/TNFα complex on tumor growth and metastasis when triggered by NIR irradiation.

## CONFLICT OF INTEREST

There are no conflicts of interest to declare. This manuscript contains original work, has not been published or presented elsewhere in part or in entirety, and is not under consideration by another journal. All study participants provided informed consent, and the study design was approved by the appropriate ethics review board. We have read and understood your journal’s policies, and we believe that neither the manuscript nor the study violates any of these.

## Supporting information


**Appendix** **S1:** Supporting Information

**Supplementary Figure 1** Structural representation of the ADP@SWNT/TNFα complex. Scheme 1. Synthesis of the Amylose Derivative Poly(L‐lysine) Dendrons (upper) and the Scheme of ADP@SWNT and the ADP@SWNT/TNFα Complex (bottom).

## Data Availability

The data that support the findings of this study are available on request from the corresponding author.
