# Supplementary material for: Application of a cationic amylose derivative loaded with single‐walled carbon nanotubes for gene delivery therapy and photothermal therapy of colorectal cancer
Source: J Biomed Mater Res A. 2022 Jan 7;110(5):1052–61. doi: 10.1002/jbm.a.37351 (PMC9302136; doi:10.1002/jbm.a.37351)
Supplement: Supplementary file 1 — Appendix S1: Supporting Information [file JBM-110-1052-s001.doc]

GCGTAATCATGGTCATAGCTGTTTCCTGTGTGAAATTGTTATCCGCTCACAATTCCACACAACATACGAG

CCGGAAGCATAAAGTGTAAAGCCTGGGGTGCCTAATGAGTGAGCTAACTCACATTAATTGCGTTGCGCTC

ACTGCCCGCTTTCCAGTCGGGAAACCTGTCGTGCCAGCTGCATTAATGAATCGGCCAACGCGCGGGGAGA

GGCGGTTTGCGTATTGGGCGCTCTTCCGCTTCCTCGCTCACTGACTCGCTGCGCTCGGTCGTTCGGCTGC

GGCGAGCGGTATCAGCTCACTCAAAGGCGGTAATACGGTTATCCACAGAATCAGGGGATAACGCAGGAAA

GAACATGTGAGCAAAAGGCCAGCAAAAGGCCAGGAACCGTAAAAAGGCCGCGTTGCTGGCGTTTTTCCAT

AGGCTCCGCCCCCCTGACGAGCATCACAAAAATCGACGCTCAAGTCAGAGGTGGCGAAACCCGACAGGAC

TATAAAGATACCAGGCGTTTCCCCCTGGAAGCTCCCTCGTGCGCTCTCCTGTTCCGACCCTGCCGCTTAC

CGGATACCTGTCCGCCTTTCTCCCTTCGGGAAGCGTGGCGCTTTCTCATAGCTCACGCTGTAGGTATCTC

AGTTCGGTGTAGGTCGTTCGCTCCAAGCTGGGCTGTGTGCACGAACCCCCCGTTCAGCCCGACCGCTGCG

CCTTATCCGGTAACTATCGTCTTGAGTCCAACCCGGTAAGACACGACTTATCGCCACTGGCAGCAGCCAC

TGGTAACAGGATTAGCAGAGCGAGGTATGTAGGCGGTGCTACAGAGTTCTTGAAGTGGTGGCCTAACTAC

GGCTACACTAGAAGAACAGTATTTGGTATCTGCGCTCTGCTGAAGCCAGTTACCTTCGGAAAAAGAGTTG

GTAGCTCTTGATCCGGCAAACAAACCACCGCTGGTAGCGGTGGTTTTTTTGTTTGCAAGCAGCAGATTAC

GCGCAGAAAAAAAGGATCTCAAGAAGATCCTTTGATCTTTTCTACGGGGTCTGACGCTCAGTGGAACGAA

AACTCACGTTAAGGGATTTTGGTCATGAGATTATCAAAAAGGATCTTCACCTAGATCCTTTTAAATTAAA

AATGAAGTTTTAAATCAATCTAAAGTATATATGAGTAAACTTGGTCTGACAGTTACCAATGCTTAATCAG

TGAGGCACCTATCTCAGCGATCTGTCTATTTCGTTCATCCATAGTTGCCTGACTCCCCGTCGTGTAGATA

ACTACGATACGGGAGGGCTTACCATCTGGCCCCAGTGCTGCAATGATACCGCGAGACCCACGCTCACCGG

CTCCAGATTTATCAGCAATAAACCAGCCAGCCGGAAGGGCCGAGCGCAGAAGTGGTCCTGCAACTTTATC

CGCCTCCATCCAGTCTATTAATTGTTGCCGGGAAGCTAGAGTAAGTAGTTCGCCAGTTAATAGTTTGCGC

AACGTTGTTGCCATTGCTACAGGCATCGTGGTGTCACGCTCGTCGTTTGGTATGGCTTCATTCAGCTCCG

GTTCCCAACGATCAAGGCGAGTTACATGATCCCCCATGTTGTGCAAAAAAGCGGTTAGCTCCTTCGGTCC

TCCGATCGTTGTCAGAAGTAAGTTGGCCGCAGTGTTATCACTCATGGTTATGGCAGCACTGCATAATTCT

CTTACTGTCATGCCATCCGTAAGATGCTTTTCTGTGACTGGTGAGTACTCAACCAAGTCATTCTGAGAAT

AGTGTATGCGGCGACCGAGTTGCTCTTGCCCGGCGTCAATACGGGATAATACCGCGCCACATAGCAGAAC

TTTAAAAGTGCTCATCATTGGAAAACGTTCTTCGGGGCGAAAACTCTCAAGGATCTTACCGCTGTTGAGA

TCCAGTTCGATGTAACCCACTCGTGCACCCAACTGATCTTCAGCATCTTTTACTTTCACCAGCGTTTCTG

GGTGAGCAAAAACAGGAAGGCAAAATGCCGCAAAAAAGGGAATAAGGGCGACACGGAAATGTTGAATACT

CATACTCTTCCTTTTTCAATATTATTGAAGCATTTATCAGGGTTATTGTCTCATGAGCGGATACATATTT

GAATGTATTTAGAAAAATAAACAAATAGGGGTTCCGCGCACATTTCCCCGAAAAGTGCCACCTGACGTCT

AAGAAACCATTATTATCATGACATTAACCTATAAAAATAGGCGTATCACGAGGCCCTTTCGTCTCGCGCG

TTTCGGTGATGACGGTGAAAACCTCTGACACATGCAGCTCCCGGAGACGGTCACAGCTTGTCTGTAAGCG

GATGCCGGGAGCAGACAAGCCCGTCAGGGCGCGTCAGCGGGTGTTGGCGGGTGTCGGGGCTGGCTTAACT

ATGCGGCATCAGAGCAGATTGTACTGAGAGTGCACCATATGCGGTGTGAAATACCGCACAGATGCGTAAG

GAGAAAATACCGCATCAGGCGCCATTCGCCATTCAGGCTGCGCAACTGTTGGGAAGGGCGATCGGTGCGG

GCCTCTTCGCTATTACGCCAGCTGGCGAAAGGGGGATGTGCTGCAAGGCGATTAAGTTGGGTAACGCCAG

GGTTTTCCCAGTCACGACGTTGTAAAACGACGGCCAGTGAATTAATTCCAAGCTAAGTGAAAGACTCTGT

CTCAAAAACCAACCTGACACCTGAGCCGGAGCGTTGCCTCTCACTACCATAAATATATATACACAACACA

CACACACACACACACACACACACACACACACACACACACACAATCATACACATAAACACAATCACACACA

CACACATGCACAATCACACACACACAATCACACACAAACATACATACACACAATCACACACAACCATACA

CACATATACACAATCACACACACAAACACAATCACACACACACACATCAATCAATCAATCAATCAATATA

CCAAAAATGATAGCTCATACCTGTAATCTTAGATGTGGGAAGGCTAAAACAGCAGGCTACCCATGACTTC

AAGGTCACCCTGGGTTACAGAGTAGCTAATAGTCTAGCTTGAACTATAGTGTAGGATCTTTAAAAAAAAA

TTAACCAACCAACCAACAAACAAACAAACAAACAACAACAACAACAAATGGAAGACAGAGGACAAAAAAA

AATAAAAGAAAAAGTAGAAAGCCAATGGAGATACTATCTTAAAATTAGAAATATTTATGACACATTCTGA

ATACTCCATGTTTATTTCAAGGAAGCCAATGTAACTTTAAGTACTATGGCCATTTAATGTAAATACTTAA

GAAAAAAAACCAAATTAATTTTGATACATGCTGCATGTGAAAGACCCCCGCTGACGGGTAGTCAATCACT

CAGAGGAGACCCTCTCAAGGAACAGCGAGACCACAAGTCGGATGCAACTGCAAGAGGGTTTATTGGATAC

ACGGGTACCCGGGCGACTCAGTCAATCGGAGGACTGGCGCCCCGAGTGAGGGGTTGTGGGCTCGAGCTCG

GGGAGCAGAAGCGCGCGAACAGAAGTCGAGCTAGCTTGCCAAACCTACAGGTGGGGTCTTTCATTCCCCC

CTTTTTCTGGAGACTAAATAAAATCTTTTATTTTATCTATGGCTCGTACTCTATAGGCTTCAGCTGGTGA

TATTGTTGAGTCAAAACTAGAGCCTGGACCACTGATATCCTGTCTTTAACAAATTGGACTAATCCGGATC

TGAAGTTCCTATACTTTCTAGAGAATAGGAACTTCGGAATAGGAACTTCGCGGCCAGATCTTTTGCGGCC

GCTTTGGGCCGGCCGAATTCTTTACTCCCTATCAGTGATAGAGAACGTATGAAGAGTTTACTCCCTATCA

GTGATAGAGAACGTATGCAGACTTTACTCCCTATCAGTGATAGAGAACGTATAAGGAGTTTACTCCCTAT

CAGTGATAGAGAACGTATGACCAGTTTACTCCCTATCAGTGATAGAGAACGTATCTACAGTTTACTCCCT

ATCAGTGATAGAGAACGTATATCCAGTTTACTCCCTATCAGTGATAGAGAACGTATAAGCTTTAGGCGTG

TACGGTGGGCGCCTATAAAAGCAGAGCTCGTTTAGTGAACCGTCAGATCGCCTGGAGCAATTCCACAACA

CTTTTGTCTTATACCAACTTTCCGTACCACTTCCTACCCTCGTAAAACCGGTCAGGTAAGTATCAAGGTT

ACAAGACAGGTTTAAGGAGACCAATAGAAACTGGGCTTGTCGAGACAGAGAAGACTCTTGCGTTTCTGAT

AGGCACCTATTGGTCTTACTGACATCCACTTTGCCTTTCTCTCCACAGGTGTCCACTCCCAGTTCAATTA

CAGCTCTTAAGGCTAGAGTACTTAATACGACTCACTATAGGCTAGCCACCATGGCTTCCAAGGTGTACGA

CCCCGAGCAACGCAAACGCATGATCACTGGGCCTCAGTGGTGGGCTCGCTGCAAGCAAATGAACGTGCTG

GACTCCTTCATCAACTACTATGATTCCGAGAAGCACGCCGAGAACGCCGTGATTTTTCTGCATGGTAACG

CTGCCTCCAGCTACCTGTGGAGGCACGTCGTGCCTCACATCGAGCCCGTGGCTAGATGCATCATCCCTGA

TCTGATCGGAATGGGTAAGTCCGGCAAGAGCGGGAATGGCTCATATCGCCTCCTGGATCACTACAAGTAC

CTCACCGCTTGGTTCGAGCTGCTGAACCTTCCAAAGAAAATCATCTTTGTGGGCCACGACTGGGGGGCTT

GTCTGGCCTTTCACTACTCCTACGAGCACCAAGACAAGATCAAGGCCATCGTCCATGCTGAGAGTGTCGT

GGACGTGATCGAGTCCTGGGACGAGTGGCCTGACATCGAGGAGGATATCGCCCTGATCAAGAGCGAAGAG

GGCGAGAAAATGGTGCTTGAGAATAACTTCTTCGTCGAGACCATGCTCCCAAGCAAGATCATGCGGAAAC

TGGAGCCTGAGGAGTTCGCTGCCTACCTGGAGCCATTCAAGGAGAAGGGCGAGGTTAGACGGCCTACCCT

CTCCTGGCCTCGCGAGATCCCTCTCGTTAAGGGAGGCAAGCCCGACGTCGTCCAGATTGTCCGCAACTAC

AACGCCTACCTTCGGGCCAGCGACGATCTGCCTAAGATGTTCATCGAGTCCGACCCTGGGTTCTTTTCCA

ACGCTATTGTCGAGGGAGCTAAGAAGTTCCCTAACACCGAGTTCGTGAAGGTGAAGGGCCTCCACTTCAG

CCAGGAGGACGCTCCAGATGAAATGGGTAAGTACATCAAGAGCTTCGTGGAGCGCGTGCTGAAGAACGAG

CAGTAAGTCGACAGATCCTACCTGCAAAGCACCAGAGGATATGGCCTCGGTGCCTCAGCATCTTCAGATG

TGCTGTCACTGTCCAATCTTCAAGCCAGCAGAGCATATGGGCTCGCTGGCTCCACTGTCATTACAGTGGA

CCTGATCTTCTCCAATCAAAGGAGCAGAGGATATGGCCTCGCTCCCTCCGACTACATCAGGCTGGAAGTG

ACATCCATCATTCATCCCAGCAGAGCATATGGGCTCGCTGGGACCACAGCATGCCTCGAAGTGCTGAGAG

ACTATCATCAAACCGACAGAGGATATGGCCTCGTCGGCTCCTACTCCCTCCTGGTGGAATACTTCTCCAC

TCTTCAACCCAGCAGAGCATATGGGCTCGCTGGGTCCATCAGAGATACAAGATCTCTCGACAGATCCTAC

CTGCAAAGCACCAGAGGATATGGCCTCGGTGCCTCAGCATCTTCAGATGTGCTGTCACTGTCCAATCTTC

AAGCCAGCAGAGCATATGGGCTCGCTGGCTCCACTGTCATTACAGTGGACCTGATCTTCTCCAATCAAAG

GAGCAGAGGATATGGCCTCGCTCCCTCCGACTACATCAGGCTGGAAGTGACATCCATCATTCATCCCAGC

AGAGCATATGGGCTCGCTGGGACCACAGCATGCCTCGAAGTGCTGAGAGACTATCATCAAACCGACAGAG

GATATGGCCTCGTCGGCTCCTACTCCCTCCTGGTGGAATACTTCTCCACTCTTCAACCCAGCAGAGCATA

TGGGCTCGCTGGGTCCATCAGAGATACAAGATCTCTCGAGCCGCGGTGCGTAAGTCTGTTAACAGAGATT

ATTTATTATTTATTTATTATTTATTTATTTACTGACACTGAACGTAATCCAGACGCGTAAGTCAGGCCGG

AAAATTCCCGCCACCGGAAGTTGAGTAGACGGTGCTGCCTGCGACTCAACCCCAGGAGGACTGGGTGAAC

AAAGCTGCGAAGTGATCCATGTAAGCCCTCAGAACCGTCTCGGAAAGAGGACCCCACATGTTGTAGCTTC

AAGGCCCAATGTCAGACCACGCCATGGCGTGCCACTCTGCGGAGAGTGCAGTCTGCGACAGTGCCCCAGG

AGGACTGGGTGAGGATCCTACCTACAAACGGCACGAGCATCAGCCGTGCCGCCCAGCATCTTCATCACTG

TCCAATCTTCAAGCGTGCACGATCACGGCACGCTCCACTGTCATTACAGTCTTATCCAATCAAACTCCAG

CAGCATCAGCGCTGGAGCTCCGACTACATCAGGACATCCATCATTCAACGCTCGACGATCACGCGAGCGT

CCACAGCATGCCTCAGACACTATCATCAAACGGCTGCACGATCACGGCAGCCGCTCCTACTGCCTCTACG

TTATCGACTCTTCTGCGACGAGCATCAGCCGTCGCAACATCGGACATACCAGATCCTACCTACAAACGGC

ACGAGCATCAGCCGTGCCGCCCAGCATCTTCATCACTGTCCAATCTTCAAGCGTGCACGATCACGGCACG

CTCCACTGTCATTACAGTCTTATCCAATCAAACTCCAGCAGCATCAGCGCTGGAGCTCCGACTACATCAG

GACATCCATCATTCAACGCTCGACGATCACGCGAGCGTCCACAGCATGCCTCAGACACTATCATCAAACG

GCTGCACGATCACGGCAGCCGCTCCTACTGCCTCTACGTTATCGACTCTTCTGCGACGAGCATCAGCCGT

CGCAACATCGGACATACCAGATCCTACCTACAAACGGCACGAGCATCAGCCGTGCCGCCCAGCATCTTCA

TCACTGTCCAATCTTCAAGCGTGCACGATCACGGCACGCTCCACTGTCATTACAGTCTTATCCAATCAAA

CTCCAGCAGCATCAGCGCTGGAGCTCCGACTACATCAGGACATCCATCATTCAACGCTCGACGATCACGC

GAGCGTCCACAGCATGCCTCAGACACTATCATCAAACGGCTGCACGATCACGGCAGCCGCTCCTACTGCC

TCTACGTTATCGACTCTTCTGCGACGAGCATCAGCCGTCGCAACATCGGACATACCAGATCCTACCTACA

AACGGCACGAGCATCAGCCGTGCCGCCCAGCATCTTCATCACTGTCCAATCTTCAAGCGTGCACGATCAC

GGCACGCTCCACTGTCATTACAGTCTTATCCAATCAAACTCCAGCAGCATCAGCGCTGGAGCTCCGACTA

CATCAGGACATCCATCATTCAACGCTCGACGATCACGCGAGCGTCCACAGCATGCCTCAGACACTATCAT

CAAACGGCTGCACGATCACGGCAGCCGCTCCTACTGCCTCTACGTTATCGACTCTTCTGCGACGAGCATC

AGCCGTCGCAACATCGGACATACCAGATCTTCAATCGATTTCGCGCGGGATCCAGACCACCTCCCCTGCG

AGCTAAGCTGGACAGCCAATGACGGGTAAGAGAGTGACATTTTTCACTAACCTAAGACAGGAGGGCCGTC

AGAGCTACTGCCTAATCCAAAGACGGGTAAAAGTGATAAAAATGTATCACTCCAACCTAAGACAGGCGCA

GCTTCCGAGGGATTTGAGATCCAGACATGATAAGATACATTGATGAGTTTGGACAAACCAAAACTAGAAT

GCAGTGAAAAAAATGCCTTATTTGTGAAATTTGTGATGCTATTGCCTTATTTGTAACCATTATAAGCTGC

AATAAACAAGTTAACAACAACAATTGCATTCATTTTATGTTTCAGGTTCAGGGGGAGGTGTGGGAGGTTT

TTTAAAGCAAGTAAAACCTCTACAAATGTGGTATGGCTGATTATGATCTGAAGTTCCTATACTATTTGAA

GAATAGGAACTTCGGAATAGGAACTTCGGATCTCATATGGCCATATGGGGGCGCCTAGAGAAGGAGTGAG

GGCTGGATAAAGGGAGGATCGAGGCGGGGTCGAACGAGGAGGTTCAAGGGGGAGAGACGGGGCGGATGGA

GGAAGAGGAGGCGGAGGCTTAGGGTGTACAAAGGGCTTGACCCAGGGAGGGGGGTCAAAAGCCAAGGCTT

CCCAGGTCACGATGTAGGGGACCTGGTCTGGGTGTCCATGCGGGCCAGGTGAAAAGACCTTGATCTTAAC

CTGGGTGATGAGGTCTCGGTTAAAGGTGCCGTCTCGCGGCCATCCGACGTTAAAGGTTGGCCATTCTGCA

GAGCAGAAGGTAACCCAACGTCTCTTCTTGACATCTACCGACTGGTTGTGAGCGATCCGCTCGACATCTT

TCCAGTGACCTAAGGTCAAACTTAAGGGAGTGGTAACAGGCTAGCCCGGGCCCATATTTTCAGACAAATA

CAGAAACACAGTCAGACAGAGACAACACAGAACGATGCTGCAGCAGACAAGACGCGCGGCGCGGCTTCGG

TCCCAAACCGAAAGCAAAAATTCAGACGGAGGCGGGAACTGTTTTAGGTTCTCGTCTCCTACCAGAACCA

CATATCCCTCCTCTAAGGGGGGTGCACCAAAGAGTCCTAAACGATCGGGATTTTAGGACTCAGGTCGGGC

CACAAAAACGGCCCCCGAAGTCCCTGGGACGTCTCCCAGGGTTGCGGCCGGGTGTTCCGAACTCGTCAGT

TCCACCACGGGTCCGCCAGATACAGAGCTAGTTAGCTAACTAGTACCGACGCAGGCGCATAAAATCAGTC

ATAGACACTAGACAATCGGACAGACACAGATAAGTTGCTGGCCAGCTTACCTCCCGGTGGTGGGTCGGTG

GTCCCTGGGCAGGGGTCTCCCGATCCCGGACGAGCCCCCAAATGAAAGACCCCCGCTGACGGGTAGTCAA

TCACTCAGAGGAGACCCTCCCAAGGAACAGCGAGACCACAAGTCGGATGCAACTGCAAGAGGGTTTATTG

GATACACGGGTACCCGGGCGACTCAGTCAATCGGAGGACTGGCGCGCCGAGTGAGGGGTTGTGGGCTCTT

TTATTGAGCTCGGGGAGCAGAAGCGCGCGAACAGAAGCGAGAAGCGAACTGATTGGTTAGTTCAAATAAG

GCACAGGGTCATTTCAGGTCCTTGGGGCACCCTGGAAACATCTGATGGTTCTCTAGAAACTGCTGAGGGC

TGGACCGCATCTGGGGACCATCTGTTCTTGGCCCTGAGCCGGGGCAGGAACTGCTTACCACAGATATCCT

GTTTGGCCCATATTCAGCTGTTCCATCTGTTCTTGGCCCTGAGCCGGGGCAGGAACTGCTTACCACAGAT

ATCCTGTTTGGCCCATATTCAGCTGTTCCATCTGTTCCTGACCTTGATCTGAACTTTTCTATTCTCAGTT

ATGTATTTTTCCATGCCTTGCAAAATGGCGTTACTTAAGCTAGCTTGCCAAACCTACAGGTGGGGTCTTT

CACATGTATATGTCAAAAATAAAAATCAACTAATTGACTAGTAATTAATATGACTGGCATAATGGGAAAT

TGATCCTGACAGATGCAAACTGGCTTCTCAGCAGCGCATTTATGTTGTCAACTGAGGAAGGAAACGTTAA

TGACAGAAACTCTAAGTAATTTCCACGTTTATCTATTTTTATTTATACTAGCTTTGGTAACAGGAATATT

GCAGCATTCATGCACATTGAAACCCTTATGAAATAAAAACATCTGTGCATTTAAAATGGAATTAACATTT

TAAATGTTAAAAAAAGCTGGCTTAGCTTCCCCCCGCCCCCTAGGGCATAGAACAAGTCAAATGCTTTATA

TATTTGAGTTTGGGATGTATTAGGAAACTCCTAAGAGCAAAGCTAGCTTG
