# Supplementary figures and images for: Application of a cationic amylose derivative loaded with single‐walled carbon nanotubes for gene delivery therapy and photothermal therapy of colorectal cancer
Source: J Biomed Mater Res A. 2022 Jan 7;110(5):1052–61. doi: 10.1002/jbm.a.37351 (PMC9302136; doi:10.1002/jbm.a.37351)

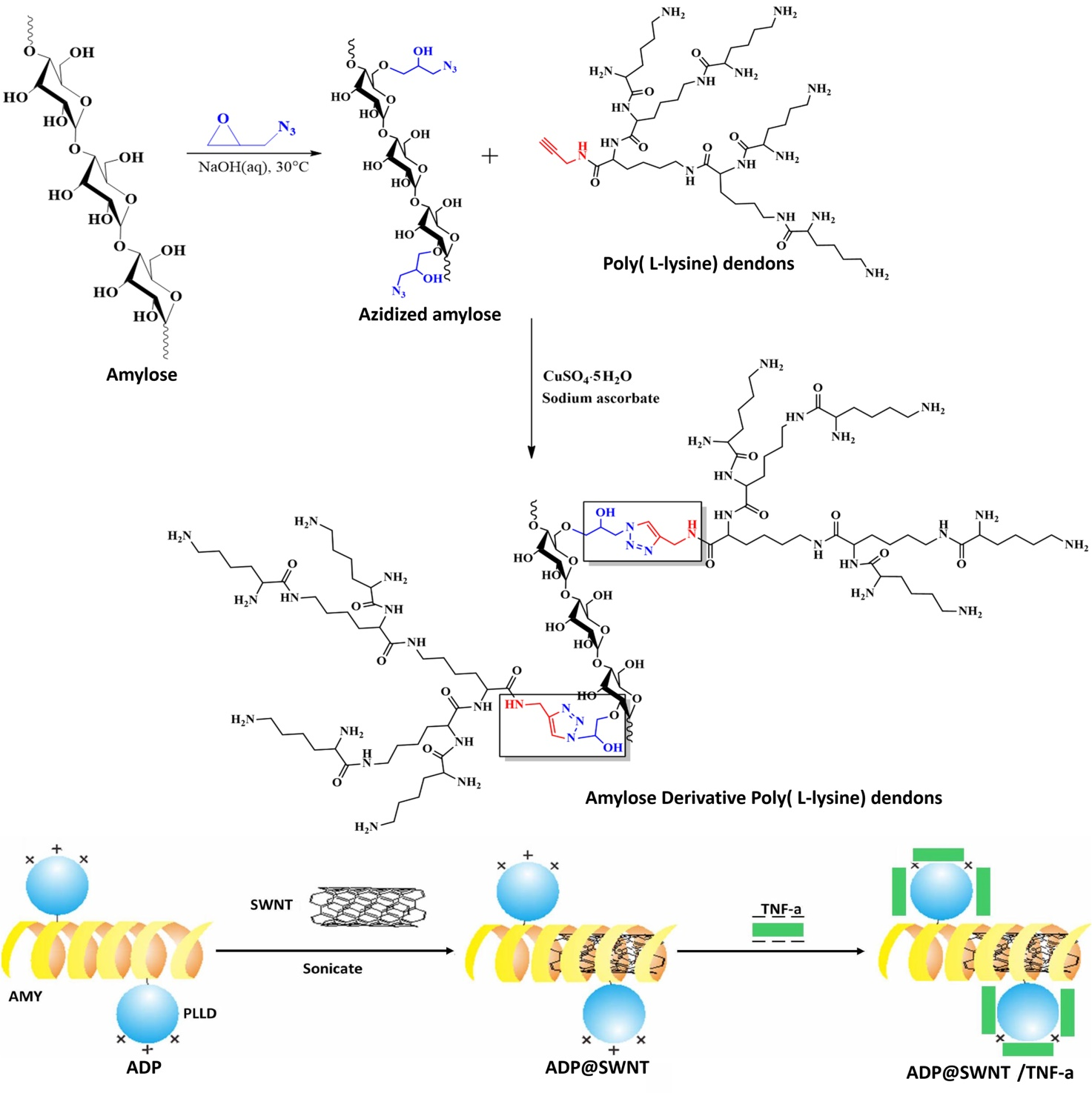

Supplement: Supplementary file 2 — Supplementary Figure 1 Structural representation of the ADP@SWNT/TNFα complex. Scheme 1. Synthesis of the Amylose Derivative Poly(L‐lysine) Dendrons (upper) and the Scheme of ADP@SWNT and the ADP@SWNT/TNFα Complex (bottom). [file JBM-110-1052-s002.tif]
